# 2,2′-[1,1′-(Hexane-1,6-diyldioxy­dinitrilo)diethyl­idyne]diphenol

**DOI:** 10.1107/S1600536808024902

**Published:** 2008-08-09

**Authors:** Wen-Kui Dong, Xue-Ni He, Yin-Xia Sun, Li Xu, Jun-Feng Tong

**Affiliations:** a School of Chemical and Biological Engineering, Lanzhou Jiaotong University, Lanzhou 730070, People’s Republic of China

## Abstract

The molecule of the title compound, C_22_H_28_N_2_O_2_, lies across an inversion centre with one half-mol­ecule in the asymmetric unit. The mol­ecule adopts an *E* configuration with respect to the azomethine C=N bond and the imino group is coplanar with the aromatic ring. Within the mol­ecule, the planar units are parallel, but extend in opposite directions from the hexa­methyl­ene bridge. There are intra­molecular O—H⋯N hydrogen bonds between the hydroxyl groups and the oxime N atoms. There are also weak inter­molecular C—H⋯O bonds that link each mol­ecule to two others, forming chains along the *a* axis.

## Related literature

For related literature, see: Atwood (1997 ▶); Canali & Sherrington (1999 ▶); Dong *et al.* (2007 ▶, 2008*a* ▶,*b* ▶,*c* ▶); Jarrahpour *et al.* (2004 ▶); Sun *et al.* (2004 ▶); Venkataramanan *et al.* (2005 ▶); Wang *et al.* (2007 ▶); Yu *et al.* (2008 ▶).
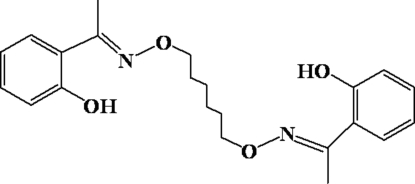


## Experimental

### 

#### Crystal data

C_22_H_28_N_2_O_4_
*M* *_r_* = 384.46
Monoclinic, 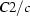

*a* = 13.0052 (19) Å
*b* = 4.6441 (6) Å
*c* = 34.221 (3) Å
β = 95.000 (2)°
*V* = 2059.0 (4) Å^3^
*Z* = 4
Mo *K*α radiation
μ = 0.09 mm^−1^
*T* = 298 (2) K
0.50 × 0.43 × 0.22 mm

#### Data collection

Bruker SMART CCD area-detector diffractometer
Absorption correction: multi-scan (*SADABS*; Sheldrick, 1996 ▶) *T*_min_ = 0.959, *T*_max_ = 0.982
4854 measured reflections
1816 independent reflections
1025 reflections with *I* > 2σ(*I*)
*R*_int_ = 0.081

#### Refinement

*R*[*F* ^2^ > 2σ(*F* ^2^)] = 0.066
*wR*(*F* ^2^) = 0.185
*S* = 1.06
1816 reflections
127 parameters
H-atom parameters constrained
Δρ_max_ = 0.15 e Å^−3^
Δρ_min_ = −0.17 e Å^−3^

### 

Data collection: *SMART* (Siemens, 1996 ▶); cell refinement: *SMART*; data reduction: *SAINT* (Siemens, 1996 ▶); program(s) used to solve structure: *SHELXS97* (Sheldrick, 2008 ▶); program(s) used to refine structure: *SHELXL97* (Sheldrick, 2008 ▶); molecular graphics: *SHELXTL* (Sheldrick, 2008 ▶); software used to prepare material for publication: *SHELXTL*.

## Supplementary Material

Crystal structure: contains datablocks global, I. DOI: 10.1107/S1600536808024902/fl2214sup1.cif

Structure factors: contains datablocks I. DOI: 10.1107/S1600536808024902/fl2214Isup2.hkl

Additional supplementary materials:  crystallographic information; 3D view; checkCIF report

## Figures and Tables

**Table 1:** Hydrogen-bond geometry (Å, °)

*D*—H⋯*A*	*D*—H	H⋯*A*	*D*⋯*A*	*D*—H⋯*A*
O2—H2⋯N1	0.82	1.83	2.549 (3)	145
C4—H4*B*⋯O2^i^	0.96	2.70	3.476 (4)	138
C11—H11⋯O2^i^	0.93	2.69	3.587 (4)	163

Symmetry code: (i) 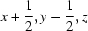.

**Fig. 1.**
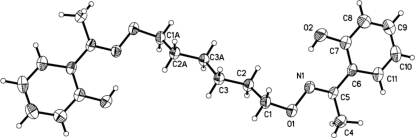
The molecular structure of the title compound with the atom numbering scheme [Symmetry codes: -x, -y + 2, -z + 1]. Displacement ellipsoids for non-hydrogen atoms are drawn at the 30% probability level.

**Fig. 2.**
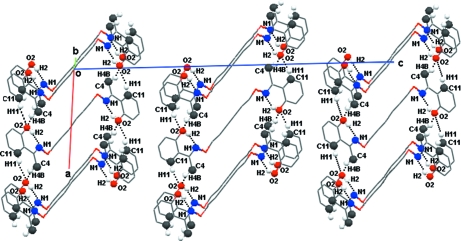
Three-dimensional packing diagram of the title compound along the b axis. Intra- and intermolecular hydrogen bonds are shown as dashed lines.

## supplementary crystallographic information

### Comment

Salen-type compounds have received a great deal of attention because of their versatility (Atwood *et al.*, 1997; Yu *et al.*, 2008; Venkataramanan *et al.*, 2005). Some, along with their transition metal complexes, exhibit anticancer and anticoagulant activity (Sun *et al.*, 2004) as well as antibacterial and antifungal properties (Jarrahpour *et al.*, 2004). In addition, some of the salen-type species can be used as catalysts, especially in the area of asymmetric catalysis (Canali *et al.*, 1999). Recently, we have reported the structures of a number molecules similar to the title compound (Wang *et al.*, 2007; Dong *et al.*, 2007, 2008*a*).

The title compound crystallized around a crystallographic inversion centre passing through the central C-C bond of the hexamethylene bridge (symmetry code: -*x*, -*y*, -*z*) to give 1/2 molecule per asymmetric unit (FIG. 1). It adopts an E configuration with respect to the azomethine C=N bond and the imino group is coplanar with the aromatic ring. Within the molecule, the planar units are parallel, but extend in opposite directions from the hexamethylene bridge. Intramolecular O(2)—H(2)···N(1) hydrogen bonds are found between the hydroxyl groups and the oxime N atoms (Table 1). This is similar to what was observed in our previously reported salen-type bisoxime compounds (Wang *et al.*, 2007; Dong *et al.*, 2007, 2008*a*).

In the unit cell there are also weak intermolecular C-H···O interactions that link each molecule to 2 others to form chains along the *a* axis (Table 1, Fig. 2),  This differs from packing interactions found for 1,1'-[(hexane-1,6-diyldioxy)bis(nitrilomethylidyne)]dinaphthalene (Dong *et al.*, 2008*b*) and 6,6'-dimethoxy-2,2'-[(hexane-1,6-diyldioxy)bis(nitrilomethylidyne)]diphenol (Dong *et al.*, 2008*c*) in which the molecules exhibit three-dimensional supramolecular structures formed through strong intermolecular π-π stacking interactions or weak intermolecular hydrogen bonds.

### Experimental

The title compound was synthesized according to an analogous method reported earlier (Dong *et al.*, 2007). To an ethanol solution (5 ml) of 2'-hydroxyacetophenone (561.4 mg, 4.00 mmol) was added an ethanol solution (4 ml) of 1,6-bis(aminooxy)hexane (296.5 mg, 2.00 mmol). The reaction mixture was stirred at 328 K for 4 h. The resulting precipitate was separated by filtration, and washed successively with ethanol and ethanol-hexane (1:4). The product was dried under vacuum to yield 434.0 mg of the title compound. Yield, 56.4%. mp. 343 K. Anal. Calc. for C_22_H_28_N_2_O_2_: C, 74.97; H, 8.01; N, 7.95. Found: C, 75.08; H, 7.85; N, 7.82.

Colorless block-shaped single crystals suitable for X-ray diffraction studies were obtained after several weeks by slow evaporation from an acetone solution.

### Refinement

Non-H atoms were refined anisotropically. H atoms were treated as riding atoms with distances C—H = 0.97 (CH_2_), 0.93 Å (CH), O—H = 0.82 Å and *U*_iso_(H) = 1.2 *U*_eq_(C) and 1.5 *U*_eq_(O).

### Crystal data

**Table:**

C_22_H_28_N_2_O_4_	*F*_000_ = 824
*M**_r_* = 384.46	*D*_x_ = 1.240 Mg m^−^^3^
Monoclinic, *C*2/*c*	Mo *K*α radiation λ = 0.71073 Å
Hall symbol: -C -2yc	Cell parameters from 1018 reflections
*a* = 13.0052 (19) Å	θ = 2.4–23.2º
*b* = 4.6441 (6) Å	µ = 0.09 mm^−^^1^
*c* = 34.221 (3) Å	*T* = 298 (2) K
β = 95.000 (2)º	Block-shaped, colorless
*V* = 2059.0 (4) Å^3^	0.50 × 0.43 × 0.22 mm
*Z* = 4	

### Data collection

**Table:**

Bruker SMART CCD area-detector diffractometer	1816 independent reflections
Radiation source: fine-focus sealed tube	1025 reflections with *I* > 2σ(*I*)
Monochromator: graphite	*R*_int_ = 0.081
*T* = 298(2) K	θ_max_ = 25.0º
φ and ω scans	θ_min_ = 2.4º
Absorption correction: multi-scan(SADABS; Sheldrick, 1996)	*h* = −12→15
*T*_min_ = 0.959, *T*_max_ = 0.982	*k* = −5→5
4854 measured reflections	*l* = −36→40

### Refinement

**Table:**

Refinement on *F*^2^	Secondary atom site location: difference Fourier map
Least-squares matrix: full	Hydrogen site location: inferred from neighbouring sites
*R*[*F*^2^ > 2σ(*F*^2^)] = 0.066	H-atom parameters constrained
*wR*(*F*^2^) = 0.185	*w* = 1/[σ^2^(*F*_o_^2^) + (0.0707*P*)^2^ + 0.7764*P*] where *P* = (*F*_o_^2^ + 2*F*_c_^2^)/3
*S* = 1.06	(Δ/σ)_max_ < 0.001
1816 reflections	Δρ_max_ = 0.16 e Å^−^^3^
127 parameters	Δρ_min_ = −0.17 e Å^−^^3^
Primary atom site location: structure-invariant direct methods	Extinction correction: none

### Special details

**Table:**

Geometry. All e.s.d.'s (except the e.s.d. in the dihedral angle between two l.s. planes) are estimated using the full covariance matrix. The cell e.s.d.'s are taken into account individually in the estimation of e.s.d.'s in distances, angles and torsion angles; correlations between e.s.d.'s in cell parameters are only used when they are defined by crystal symmetry. An approximate (isotropic) treatment of cell e.s.d.'s is used for estimating e.s.d.'s involving l.s. planes.
Refinement. Refinement of *F*^2^ against ALL reflections. The weighted *R*-factor *wR* and goodness of fit *S* are based on *F*^2^, conventional *R*-factors *R* are based on *F*, with *F* set to zero for negative *F*^2^. The threshold expression of *F*^2^ > σ(*F*^2^) is used only for calculating *R*-factors(gt) *etc*. and is not relevant to the choice of reflections for refinement. *R*-factors based on *F*^2^ are statistically about twice as large as those based on *F*, and *R*- factors based on ALL data will be even larger.

### Fractional atomic coordinates and isotropic or equivalent isotropic displacement parameters (Å^2^)

**Table:**

	*x*	*y*	*z*	*U*_iso_*/*U*_eq_	
N1	0.20750 (18)	0.7620 (6)	0.39536 (6)	0.0558 (7)	
O1	0.25649 (15)	0.9218 (5)	0.42661 (6)	0.0689 (7)	
O2	0.04940 (15)	0.5889 (6)	0.35249 (6)	0.0814 (8)	
H2	0.0805	0.6818	0.3702	0.122*	
C1	0.1805 (2)	1.0874 (8)	0.44434 (8)	0.0634 (9)	
H1A	0.1380	1.1879	0.4240	0.076*	
H1B	0.2149	1.2310	0.4614	0.076*	
C2	0.1115 (2)	0.9044 (7)	0.46802 (8)	0.0568 (8)	
H2A	0.0712	0.7748	0.4505	0.068*	
H2B	0.1541	0.7885	0.4866	0.068*	
C3	0.0392 (2)	1.0874 (7)	0.49000 (7)	0.0545 (8)	
H3A	0.0796	1.2005	0.5096	0.065*	
H3B	0.0030	1.2202	0.4717	0.065*	
C4	0.3827 (2)	0.5826 (10)	0.38989 (9)	0.0837 (12)	
H4A	0.4084	0.7690	0.3980	0.126*	
H4B	0.4184	0.5160	0.3682	0.126*	
H4C	0.3938	0.4498	0.4113	0.126*	
C5	0.2690 (2)	0.6031 (7)	0.37758 (8)	0.0501 (7)	
C6	0.2230 (2)	0.4313 (7)	0.34493 (7)	0.0496 (7)	
C7	0.1174 (2)	0.4300 (8)	0.33391 (8)	0.0606 (8)	
C8	0.0770 (3)	0.2574 (9)	0.30335 (9)	0.0776 (10)	
H8	0.0062	0.2574	0.2965	0.093*	
C9	0.1397 (3)	0.0870 (9)	0.28307 (9)	0.0786 (11)	
H9	0.1116	−0.0283	0.2626	0.094*	
C10	0.2445 (3)	0.0870 (8)	0.29310 (9)	0.0751 (10)	
H10	0.2876	−0.0278	0.2794	0.090*	
C11	0.2846 (2)	0.2562 (8)	0.32321 (8)	0.0626 (9)	
H11	0.3556	0.2553	0.3296	0.075*	

### Atomic displacement parameters (Å^2^)

**Table:**

	*U*^11^	*U*^22^	*U*^33^	*U*^12^	*U*^13^	*U*^23^
N1	0.0568 (14)	0.0595 (17)	0.0530 (13)	−0.0017 (15)	0.0155 (11)	−0.0068 (13)
O1	0.0583 (12)	0.0827 (17)	0.0685 (12)	−0.0034 (13)	0.0215 (10)	−0.0199 (12)
O2	0.0554 (13)	0.093 (2)	0.0976 (16)	0.0061 (14)	0.0143 (12)	−0.0252 (15)
C1	0.0693 (19)	0.059 (2)	0.0651 (17)	−0.0062 (18)	0.0267 (15)	−0.0163 (16)
C2	0.0571 (17)	0.056 (2)	0.0589 (16)	0.0034 (17)	0.0163 (13)	−0.0037 (16)
C3	0.0593 (17)	0.0524 (19)	0.0535 (16)	0.0009 (16)	0.0144 (13)	−0.0038 (15)
C4	0.0543 (19)	0.108 (3)	0.089 (2)	0.011 (2)	0.0101 (16)	−0.027 (2)
C5	0.0506 (16)	0.0483 (18)	0.0542 (15)	0.0036 (15)	0.0202 (13)	0.0017 (15)
C6	0.0562 (17)	0.0486 (18)	0.0464 (14)	0.0036 (16)	0.0172 (12)	0.0078 (14)
C7	0.0634 (19)	0.059 (2)	0.0610 (18)	0.0055 (18)	0.0157 (15)	0.0015 (17)
C8	0.077 (2)	0.079 (3)	0.077 (2)	−0.004 (2)	0.0061 (18)	−0.007 (2)
C9	0.108 (3)	0.070 (3)	0.0583 (19)	−0.003 (2)	0.008 (2)	−0.0042 (18)
C10	0.107 (3)	0.064 (2)	0.0571 (19)	0.019 (2)	0.0239 (18)	−0.0010 (18)
C11	0.074 (2)	0.061 (2)	0.0547 (17)	0.012 (2)	0.0190 (15)	0.0036 (17)

### Geometric parameters (Å, °)

**Table:**

N1—C5	1.281 (3)	C4—H4A	0.9600
N1—O1	1.408 (3)	C4—H4B	0.9600
O1—C1	1.429 (3)	C4—H4C	0.9600
O2—C7	1.352 (4)	C5—C6	1.458 (4)
O2—H2	0.8200	C6—C7	1.393 (4)
C1—C2	1.520 (4)	C6—C11	1.399 (4)
C1—H1A	0.9700	C7—C8	1.384 (5)
C1—H1B	0.9700	C8—C9	1.368 (5)
C2—C3	1.515 (4)	C8—H8	0.9300
C2—H2A	0.9700	C9—C10	1.376 (5)
C2—H2B	0.9700	C9—H9	0.9300
C3—C3^i^	1.512 (5)	C10—C11	1.363 (5)
C3—H3A	0.9700	C10—H10	0.9300
C3—H3B	0.9700	C11—H11	0.9300
C4—C5	1.504 (4)		
			
C5—N1—O1	113.9 (2)	C5—C4—H4C	109.5
N1—O1—C1	108.8 (2)	H4A—C4—H4C	109.5
C7—O2—H2	109.5	H4B—C4—H4C	109.5
O1—C1—C2	112.8 (3)	N1—C5—C6	116.6 (2)
O1—C1—H1A	109.0	N1—C5—C4	122.9 (3)
C2—C1—H1A	109.0	C6—C5—C4	120.5 (3)
O1—C1—H1B	109.0	C7—C6—C11	116.8 (3)
C2—C1—H1B	109.0	C7—C6—C5	122.6 (3)
H1A—C1—H1B	107.8	C11—C6—C5	120.6 (3)
C3—C2—C1	111.8 (2)	O2—C7—C8	116.8 (3)
C3—C2—H2A	109.3	O2—C7—C6	122.7 (3)
C1—C2—H2A	109.3	C8—C7—C6	120.5 (3)
C3—C2—H2B	109.3	C9—C8—C7	120.9 (3)
C1—C2—H2B	109.3	C9—C8—H8	119.5
H2A—C2—H2B	107.9	C7—C8—H8	119.5
C3^i^—C3—C2	113.3 (3)	C8—C9—C10	119.6 (3)
C3^i^—C3—H3A	108.9	C8—C9—H9	120.2
C2—C3—H3A	108.9	C10—C9—H9	120.2
C3^i^—C3—H3B	108.9	C11—C10—C9	119.6 (3)
C2—C3—H3B	108.9	C11—C10—H10	120.2
H3A—C3—H3B	107.7	C9—C10—H10	120.2
C5—C4—H4A	109.5	C10—C11—C6	122.5 (3)
C5—C4—H4B	109.5	C10—C11—H11	118.8
H4A—C4—H4B	109.5	C6—C11—H11	118.8
			
C5—N1—O1—C1	−179.4 (2)	C5—C6—C7—O2	0.3 (5)
N1—O1—C1—C2	72.4 (3)	C11—C6—C7—C8	1.3 (5)
O1—C1—C2—C3	173.9 (2)	C5—C6—C7—C8	−178.3 (3)
C1—C2—C3—C3^i^	173.2 (3)	O2—C7—C8—C9	−179.4 (3)
O1—N1—C5—C6	179.9 (2)	C6—C7—C8—C9	−0.7 (5)
O1—N1—C5—C4	1.9 (4)	C7—C8—C9—C10	−0.1 (5)
N1—C5—C6—C7	−1.9 (4)	C8—C9—C10—C11	0.2 (5)
C4—C5—C6—C7	176.1 (3)	C9—C10—C11—C6	0.5 (5)
N1—C5—C6—C11	178.5 (3)	C7—C6—C11—C10	−1.2 (4)
C4—C5—C6—C11	−3.4 (4)	C5—C6—C11—C10	178.4 (3)
C11—C6—C7—O2	179.9 (3)		

Symmetry codes: (i) −*x*, −*y*+2, −*z*+1.

### Hydrogen-bond geometry (Å, °)

**Table:**

*D*—H···*A*	*D*—H	H···*A*	*D*···*A*	*D*—H···*A*
O2—H2···N1	0.82	1.83	2.549 (3)	145
C4—H4B···O2^ii^	0.96	2.70	3.476 (4)	138
C11—H11···O2^ii^	0.93	2.69	3.587 (4)	163

Symmetry codes: (ii) *x*+1/2, *y*−1/2, *z*.
